# The impact of environment on genetic and epigenetic variation in *Trifolium pratense* populations from two contrasting semi-natural grasslands

**DOI:** 10.1098/rsos.211406

**Published:** 2022-05-18

**Authors:** Theresa Anna Lehmair, Peter Poschlod, Christoph Reisch

**Affiliations:** Department of Ecology and Conservation Biology, University of Regensburg, Institute of Plant Sciences, 93053 Regensburg, Germany

**Keywords:** epigenetic variation, genetic variation, environmental conditions, *Trifolium pratense*

## Abstract

Central European grasslands, such as calcareous grasslands and oat-grass meadows, are characterized by diverse environmental conditions and management regimes. Therefore, we aimed to determine potential differences in genetic and epigenetic variation patterns between the contrasting habitats and to identify the drivers of genetic and epigenetic variation. We investigated the genetic and epigenetic variation of the ecologically variable plant species *Trifolium pratense* L. applying amplified fragment length polymorphism and methylation-sensitive amplification polymorphism analyses. We observed low levels of genetic and epigenetic differentiation among populations and between habitat types. Genetic and epigenetic variations were not interdependent. Thus, genetic variation was significantly isolated by habitat dissimilarity, whereas epigenetic variation was affected by environment. More specifically, we observed a significant correlation of epigenetic diversity with soil moisture and soil pH (the latter potentially resulting in phosphorus limitation). Genetic variation was, therefore, affected more strongly by habitat-specific environmental conditions induced by land use-related disturbance and gene flow patterns, while epigenetic variation was driven by challenging environmental conditions.

## 1. Introduction

Hutchinson [1] defined the concept of ‘habitat’ as a collection of environmental conditions allowing a plant species to survive and to grow. Applying this definition, each habitat represents a specific environmental setting with certain selective pressures [2]. Plant species need to respond to specific soil or climatic conditions to cope with these pressures. Furthermore, they are subjected to different management regimes in anthropogenic habitats, such as semi-natural grasslands. Type, intensity, and time of management may cause large differences in the plant composition of Central European grasslands. Mowing, for instance, happens abruptly and affects all species simultaneously, while more continuously grazing never pertains a population on the whole [3]. Widespread and common species such as *Trifolium pratense* often have a very broad ecological niche and may occur only with specific adaptations in grassland types of different ecological conditions and management. For instance, *T. pratense* occurs in both calcareous grasslands (CGs) and oat-grass meadows (OMs). Semi-natural CGs developed from grazing over thousands of years and represent nutrient-poor grasslands with relative dry soil conditions [4]. However, comparative nutrient-rich, mesic OMs developed from cutting for hay making since the Middle Ages [5]. Although both habitats often occur within the same geographic region, they reveal contrasting environmental conditions. Therefore, *T. pratense* in CGs and OMs appeared as a promising model system for studying genetic and epigenetic variation patterns within contrasting habitats.

Previous studies showed that environmental conditions may affect the genetic code of a plant species indirectly [6–8] indicating that the reaction of a plant species to changing environmental conditions is exclusively based on genetic variation [2]. During the last few decades, numerous studies demonstrated that plant species can react to diverse environments without changing their DNA sequence (e.g. [2,7–11]). These metastable, but mostly heritable changes in gene expression are induced by chemical DNA and histone modifications as well as interference by small non-coding RNAs [12].

The potential reversible DNA methylation of cytosine represents the most studied epigenetic mechanism with important effects on ecologically relevant traits [13,14]. Cytosine methylations occur throughout the genome in all sequence contexts [15] and are predominantly located in repetitive sequences and transposable elements [16]. From there, cytosine methylations could regulate transposon silencing and gene expression without changing the underlying genetic code [17]. Methylation-sensitive amplification polymorphism (MSAP) analyses, established by Schulz *et al*. [16], allow the identification of methylation-based epiallelic markers in natural populations of non-model plants [18]. These markers enable a genome-wide snapshot of epigenetic variation. Nevertheless, information about their role in natural populations is still scarce [13], since only a few studies have addressed the impact of epigenetic variation on genetically diverse, non-model plant species so far [2,9,18,19].

Changes in DNA methylation were observed to increase in response to biotic and abiotic stressors [20–22] such as herbivores [23], salinity [13], drought [24], extreme temperatures, or nutrient limitation [25]. DNA methylation alterations, caused by challenging environmental conditions, are common, sequence-independent, readily generated, and mostly heritable [22]. Thus, epigenetic variation, provoked by DNA methylation, provides a valuable tool for plant species to rapidly adapt and survive under challenging environmental conditions [26]. Hereby, different challenging environmental conditions may induce hypo- or hypermethylation or shifts in global methylation patterns depending on plant species or rather genotype [12,22,24].

During the last few decades, numerous studies on various plant species observed profound effects of environmental conditions on both genetic and epigenetic variation patterns (e.g. [4–7,9,25]). Thus, most plant species are diverse as a result of complex interactions between genetic, epigenetic, and environmental variation [27]. Previous studies stated a certain correlation of genetic and epigenetic variation [9,19]. Hence, epigenetic variation may be controlled by the underlying genetic code [28], but environmental parameters can also directly change epigenetic variation [29]. In the studies mentioned above, epigenetic differentiation was, therefore, generally more closely related to environment than to genetic differentiation. Thus, they indicate that heritable epigenetic changes might constitute a key variable for local adaptation [27]. Therefore, genetic and epigenetic variation should be tested for interdependence when considering the impact of environmental factors on genetic and epigenetic variation.

We asked the following questions to gain a better understanding about the impact of contrasting environmental conditions on genetic and epigenetic variation in *T. pratense*: (i) are populations genetically and/or epigenetically differentiated among contrasting grassland habitats or are they isolated by distance? (ii) does genetic and/or epigenetic diversity differ between CG and OM populations? (iii) what is the impact of environment on genetic and/or epigenetic diversity levels? and (iv) is genetic and epigenetic variation of *T. pratense* populations interdependent?

## 2. Methods

### 2.1. Study design

**Figure 1.**
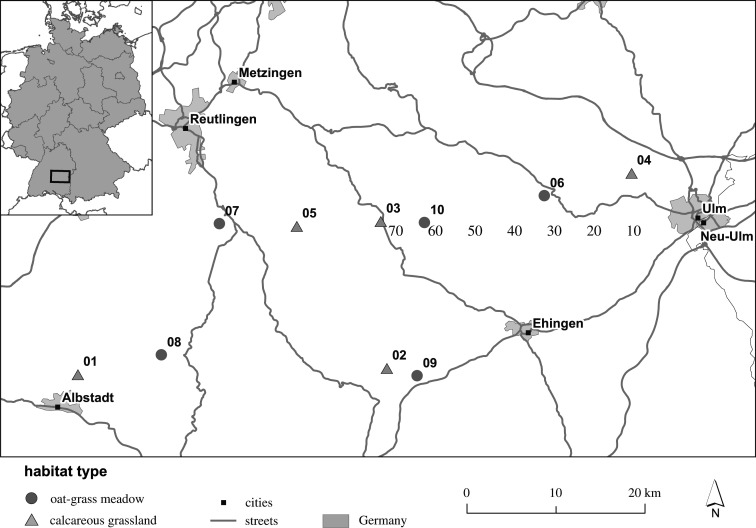
Geographic position of the analysed populations on calcareous grasslands (triangles) and oat-grass meadows (points), five each. This map was created using the software ArcGIS® 10.3.1 (Esri, Redlands, CA, USA).

**Figure 2.**
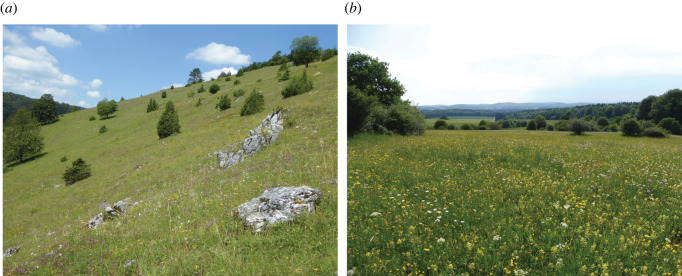
Semi-natural calcareous grassland (*a*) and oat-grass meadow (*b*).

For our study, we selected CGs and OMs, five each, all over the Swabian Alb in southwest Germany (figure 1; electronic supplementary material, table S1). Semi-natural CGs (figure 2) on the Swabian Alb are characterized by steep slopes, shallow soils, and relatively dry soil conditions [30]. They are mainly grazed by sheep from late spring until early summer [3]. Continuous, selective grazing and physical disturbance by trampling impoverish soil nutrients and shape the heterogeneous soil and sward structure of this habitat type [31]. Oat-grass (*Arrhenatherum elatius* (L.) J. Presl & C. Presl) meadows (figure 2) are traditionally managed with two (or three) cuttings per year. Manure and more recently mineral fertilizer are applied to maintain productivity [5,32]. These lowland hay meadows show a more unified soil and sward structure than CGs, since mowing affects all species simultaneously and in the same way [33]. Both habitats reveal contrasting environmental conditions although they are located nearby each other within the same geographic region. Therefore, CGs and OMs of this region appeared as a promising model system for studying genetic and epigenetic variation patterns.

The widespread species *T. pratense* L. occurs in CGs and OMs. Therefore, it represents an appropriate model organism to analyse genetic and epigenetic variation within these contrasting habitats. The red clover (Fabaceae, 2*n* = 14) flowers between June and September [34]. It is self-sterile and, therefore, nearly exclusively pollinated by bumblebees [35]. The persistent seeds may survive at least 39 years within the soil seed bank [36]. *T. pratense* is an essential species for profitable grassland management due to its high fodder value [37] and its ability to improve soil properties by nitrogen fixation [38].

Ellenberg indicator values (EIVs), using plants as bio-indicators, were applied to gain information about environmental conditions (electronic supplementary material, table S2). Environmental conditions may often fluctuate in time and space and can, thus, not be estimated in a single measurement [39]. The indicator values have advantages over conducting measurements [40], since plants represent the integrated expression of the values of those environmental variables. Furthermore, measurements rely on technical equipment and often need more time and financial effort than floristic observations. EIVs, established by Ellenberg *et al*. [41], represent the realized optima of a species. They are expressed as ordinal numbers reflecting the species' requirements along, for example, light, soil moisture, soil reaction/pH, soil nitrogen, soil salinity, or temperature gradients. The availability of light, nutrients as well as soil moisture and pH represent the local environmental conditions of a habitat [33]. EIVs show some limitations since they were defined based on observations of species’ occurrences of different sites and not from systematic measurements. Nevertheless, EIVs appear to reflect habitat quality well for Central European grasslands [39]. Therefore, we calculated the mean weighted light, soil moisture, soil reaction/pH, and soil nitrogen EIVs per study site using the species' abundance from previously conducted vegetation surveys (unpublished data) as described by Diekmann [39]. These indicator values will be named simply as light, soil moisture, soil pH, and soil nitrogen throughout this study.

For molecular analyses, we identified the species in the field and took leaf samples from 16 individuals per population and species to cover more than 90% of the total (epi)genetic diversity [42]. Samples were collected in June and July 2016 and 2017. They were dried and stored on silica gel at room temperature until DNA extraction.

### 2.2. Genetic and epigenetic fingerprinting

All 160 individuals were analysed genetically and epigenetically. DNA was extracted following the CTAB protocol from Rogers & Bendich [43] modified by Reisch [44]. A spectrophotometer was used to measure DNA quality and concentration. All DNA samples were diluted to the same level of 7.8 ng DNA per µl H_2_O.

Genetic variation within populations was determined using genome-wide genotyping with amplified fragment length polymorphisms (AFLPs) [45]. The AFLP analyses were performed following the standardized protocol of Beckmann Coulter [46,47]. After a screening of 42 primer combinations, we selected three appropriate combinations for the selective amplification (electronic supplementary material, table S3).

MSAP analyses were performed in accordance to the technique of Schulz *et al.* [16]. Thus, MSAP analyses follow the protocol of modified AFLP analyses replacing the frequent cutter *MSeI* by two isoschizomers *HpaII* and *MspI*. These restriction enzymes attach at the same tetranucleotide (5′ CCGG) sequence with differing sensitivity to cytosine methylation states and cover, thus, the most frequent methylation types in the CG and CHG (with H = A, C or T) sequence context [15,16]. Therefore, they allow the comparison of large amounts of anonymous, methylation-sensitive CCGG regions across the genome for a large number of individuals [16]. Thirty-six primer combinations were screened to identify three suitable combinations for the selective amplification (electronic supplementary material, table S3).

The fluorescence-labelled DNA fragments were separated by capillary gel electrophoresis using an automated capillary electrophoresis machine (GeXP, Beckmann Coulter). Ninety-six samples and 48 individuals (*HpaII* and *MspI*) were analysed per run, respectively. Samples without a clear banding pattern between 60 and 420 bp were repeated and only strong and clearly defined fragments were taken into account for further analyses. Fragment data were analysed manually with the software Bionumerics 7.6.2 (Applied Maths, Kortrijk, Belgium).

After fragment detection, we applied the ‘mixed scoring 2’ by Schulz *et al.* [16] to score the presence–absence matrices for MSAP fragments. Schulz *et al*. [16] defined four conditions for the resulting *EcoRI*/*HpaII* and *EcoRI*/*MspI* fragment profiles: (i) fragments are present in both profiles (unmethylated state/u-type), (ii) fragments are present only in *EcoRI*/*MspI* profiles (hemi- or fully methylated at the internal cytosine/m-type), (iii) fragments are present only in *EcoRI*/*HpaII* profiles (hemimethylated at the external cytosine/h-type), and (iv) complete absence of fragments in both profiles (uninformative state).

The reproducibility of the AFLP and MSAP analyses was tested by calculating the genotyping error rate [48]. Ten per cent of all analysed samples were replicated twice and the percentage of fragments with differences between original and replicate was evaluated. The genotyping error rates for AFLP analyses were 5.24% and for MSAP analyses were 1.02%.

### 2.3. Data analyses

Genetic and epigenetic differentiation within and among populations as well as between habitat types were partitioned with hierarchical analyses of molecular variance (AMOVA). AMOVAs were calculated based on pairwise Euclidean distances among samples using the software GenAlEx 6.41 [49].

Epigenetic and genetic distance matrices were calculated within the AMOVA (*Φ*_PT_ values; electronic supplementary material, table S4). Pairwise geographic distances (km) were calculated from coordinates, and for habitat types, a habitat dissimilarity matrix was constructed by coding pairs of CG/OM populations by ‘1′ and pairs of equal habitats by ‘0’ (electronic supplementary material, table S5). A correlation between genetic and epigenetic distance matrices was examined applying a simple Mantel test. Geographic and habitat dissimilarity matrices were also checked for correlation patterns. Genetic and epigenetic isolation by distance (IBD) and isolation by habitat dissimilarity (IBH) were tested performing simple and partial Mantel tests with 9999 permutations applying the ‘vegan’ library in R [50].

Although simple and partial Mantel tests are suitable to test dissimilarity hypotheses [51,52], e.g. for IBD, they have been criticized as showing inflated type I error and low statistical power [52–54]. Since the controversy on their validity in hypothesis testing remains unresolved [55], Wang's [56] method based on multiple matrix regression with randomization (MMRR) was additionally performed. Instead of correlation analyses with removed effects of geography or habitat dissimilarity, this method simultaneously applies the effects of geographic distance and habitat dissimilarity on genetic or epigenetic distance matrices. Distance matrices were scaled and centred to obtain comparable standardized linear regression coefficients [55] before using the MMRR function of Wang [56] available from the Dryad Data Repository (doi:10.5061/dryad.kt71r).

Genetic and epigenetic diversity within populations were determined using the R script ‘MSAP_calc’ [16]. Applying the function ‘descriptive_parameters', (i) percentage of total and private bands, (ii) percentage of polymorphic loci and subepiloci, and (iii) mean Shannon's information index was calculated with SI = −∑*p*_i_ · log_2_*p*_i_, where *p*_i_ is the frequency of the (epi)genetic marker score ‘1’ within the population. The acronyms ‘SI_gen_’ and ‘SI_epigen_’ stand for the mean Shannon's information index and will be substituted by the terms ‘genetic diversity’ and ‘epigenetic diversity’ in the discussion.

Two-sided T-tests (and Wilcoxon–Mann–Whitney tests if necessary) were calculated to examine differences of SI_gen_, SI_epigen_, and environmental parameters (light, soil moisture, soil pH, and soil nitrogen) between CG and OM populations.

Possible correlations of SI_gen_ and SI_epigen_ with light, soil moisture, soil pH, and soil nitrogen were analysed with correlation tests (Pearson correlation coefficients) applying the ‘PerformanceAnalytics' [57] and ‘Hmisc’ [58] libraries in R.

Differences between SI_gen_ and SI_epigen_ were examined with paired T-tests. Additionally, SI_gen_ and SI_epigen_ were tested for interdependence applying the correlation tests as mentioned above. Unless otherwise stated, the R environment [59] was used for statistical analyses.

## 3. Results

### 3.1. Genetic and epigenetic differentiation

Hierarchical AMOVA of genetic data (table 1) revealed a global *Φ*_PT_ of 0.07 with a differentiation between habitat types of 3% and a differentiation among populations of 4%. The hierarchical AMOVA of the combined epigenetic dataset resulted with 0.05 in a lower *Φ*_PT_. 1% of epigenetic variance resided between habitat types and 4% among populations. Values of epigenetic differentiation for h-, m- and u-subepiloci are given in table 1.

**Table 1.** Genetic and epigenetic variation among populations of different habitat types, among and within studied populations. AMOVA; *p*-values were calculated with 999 iteration steps; ***p ≤ 0.001. d.f., degree of freedom; SS, sum of squares; MS, mean squares; Est. Var., estimated variation; %, the proportion of genetic variation. h-subepiloci, CHG-hemimethylated; m-subepiloci, CG-methylated; u-subepiloci, non-methylated.

	AMOVA	d.f.	SS	MS	Est. Var.	%	*Φ*_PT_
AFLP loci (*n* = 124)	among habitats	1	46.54	46.54	0.36	3	0.070***
	among populations	8	140.50	17.56	0.44	4	
	within populations	150	1584.44	10.56	10.56	93	
MSAP							
all subepiloci (*n* = 408)	among habitats	1	109.89	109.89	0.42	1	0.050***
	among populations	8	608.23	76.03	1.93	4	
	within populations	150	6767.38	45.12	45.12	95	
h-subepiloci (*n* = 116)	among habitats	1	24.20	24.20	0.16	2	0.080***
	among populations	8	92.50	11.56	0.35	6	
	within populations	150	885.88	5.91	5.91	92	
m-subepiloci (*n* = 144)	among habitats	1	38.63	38.63	0.11	1	0.039***
	among populations	8	240.40	30.05	0.67	3	
	within populations	150	2897.25	19.32	19.32	96	
u-subepiloci (*n* = 148)	among habitats	1	47.06	47.06	0.16	1	0.051***
	among populations	8	275.33	34.42	0.91	4	
	within populations	150	2984.25	19.90	19.90	95	

A simple Mantel test revealed no correlation between genetic and epigenetic differentiation across all populations (*r* = 0.30; *p* = 0.069). Geographic distance (IBD) and habitat dissimilarity (IBH) were also not correlated (*r* = −0.09; *p* = 0.776).

Simple and partial Mantel tests as well as MMRR revealed no significant relationship between genetic or epigenetic differentiation and geographic distance (IBD) (*p* > 0.05; tables 2 and 3). However, genetic differentiation correlated significantly with habitat dissimilarity (IBH) in simple (*r* = 0.51; *p* = 0.004) and partial (*r* = 0.50; *p* = 0.003) Mantel tests (table 2) as well as MMRR (*r* = 0.02; *p* = 0.010) (table 3). Epigenetic differentiation showed no correlation with habitat dissimilarity (IBH) (*p* > 0.05; tables 2 and 3).

**Table 2.** Simple and partial Mantel tests for genetic and epigenetic pairwise population *Φ*_PT_ with geographic distance (km) and habitat dissimilarity matrices. *P*-values were calculated with 9999 permutations. h-subepiloci, CHG-hemimethylated; m-subepiloci, CG-methylated; u-subepiloci, non-methylated.

	geographic distance matrix				habitat dissimilarity distance matrix			
	simple test		partialled on habitat dissimilarity		simple test		partialled on geographic distance	
	*r*	*p*	*r*	*p*	*r*	*p*	*r*	*p*
AFLP	−0.08	0.652	−0.04	0.571	0.51	0.004	0.50	0.003
MSAP								
all subepiloci	−0.16	0.795	−0.14	0.767	0.20	0.113	0.19	0.120
h-subepiloci	−0.11	0.686	−0.09	0.653	0.22	0.099	0.21	0.108
m-subepiloci	−0.22	0.896	−0.21	0.880	0.12	0.237	0.10	0.273
u-subepiloci	−0.02	0.540	0.00	0.504	0.18	0.142	0.18	0.135

**Table 3.** Summary of MMRR relating genetic and epigenetic distance matrices (*Φ*_PT_) with geographic (km) and habitat dissimilarity distance matrices. *P*-values were calculated with 9999 permutations. h-subepiloci, CHG-hemimethylated; m-subepiloci, CG-methylated; u-subepiloci, non-methylated.

differentiation matrix	overall regression		linear predictor matrices			
			geographic distance		habitat dissimilarity	
	*F*	*p*	coefficient	*p*	coefficient	*p*
AFLP	10.93	0.014	0.001	0.698	0.015	0.010
MSAP						
all subepiloci	2.10	0.189	−0.002	0.566	0.004	0.017
h-subepiloci	2.73	0.139	−0.004	0.599	0.011	0.015
m-subepiloci	1.58	0.299	−0.004	0.373	0.002	0.143
u-subepiloci	1.27	0.342	0.001	0.845	0.004	0.055

### 3.2. Genetic and epigenetic diversity

A total of 159 MSAP fragments were analysed and scoring revealed 408 markers consisting of 116 CHG-hemimethylated h-epiloci, 144 CG-methylated m-epiloci, and 148 non-methylated u-epiloci. Generally, epigenetic diversity across populations showed mean values of 73.7% bands per population, 0.8% private bands, 69.3% polymorphic subepiloci and a mean Shannon's information index (SI_epigen_) of 0.46 (table 4). Further values of epigenetic diversity for h-, m- and u-subepiloci are given in table 4.

**Table 4.** Genetic and epigenetic diversity measures within calcareous grassland and oat-grass meadow populations of *T. pratense*. 1–5, calcareous grassland populations; 6–10, oat-grass meadow populations. h-subepiloci, CHG-hemimethylated; m-subepiloci, CG-methylated; u-subepiloci, non-methylated.

	AFLP	MSAP all	MSAP h-subepiloci	MSAP m-subepiloci	MSAP u-subepiloci
number of loci	124	408	116	144	148
bands per population (%)					
1	99.2	71.8	42.2	84.7	82.4
2	98.4	71.3	39.7	82.6	85.1
3	98.4	69.4	43.1	81.3	78.4
4	100.0	72.5	44.8	78.5	88.5
5	99.2	76.0	52.6	84.0	86.5
6	97.6	74.3	46.6	84.0	86.5
7	97.6	81.6	60.3	88.2	91.9
8	97.6	70.1	33.6	82.6	86.5
9	99.2	75.2	47.4	81.9	90.5
10	99.2	74.5	42.2	83.3	91.2
mean	98.6	73.7	45.3	83.1	86.8
s.e.	0.3	1.1	2.3	0.8	1.3
private bands per population (%)					
1	0.0	1.7	6.0	0.0	0.0
2	0.0	0.5	0.9	0.7	0.0
3	0.0	1.0	2.6	0.7	0.0
4	0.0	0.5	1.7	0.0	0.0
5	0.0	0.5	0.9	0.7	0.0
6	0.0	0.0	0.0	0.0	0.0
7	0.0	1.0	2.6	0.7	0.0
8	0.0	1.0	2.6	0.0	0.7
9	0.0	1.2	2.6	0.7	0.7
10	0.0	0.7	2.6	0.0	0.0
mean	0.0	0.8	2.2	0.3	0.1
s.e.	0.0	0.2	0.5	0.1	0.1
percentage of polymorphic loci					
1	46.8	68.1	42.2	79.2	77.7
2	52.4	68.1	39.7	79.2	79.7
3	50.0	64.0	43.1	73.6	71.0
4	50.8	67.7	44.8	75.0	78.4
5	48.4	71.8	52.6	78.5	80.4
6	45.2	69.1	46.6	79.9	76.4
7	54.0	78.4	60.3	85.4	85.8
8	46.8	65.4	33.6	79.9	76.4
9	50.0	71.6	47.4	79.9	82.4
10	51.6	69.1	42.2	78.5	81.1
mean	49.6	69.3	45.3	78.9	78.9
s.e.	0.9	1.3	2.3	1.0	1.3
SI					
1	0.36	0.45	0.23	0.54	0.54
2	0.40	0.45	0.22	0.55	0.55
3	0.36	0.42	0.21	0.52	0.50
4	0.35	0.43	0.22	0.51	0.52
5	0.34	0.49	0.26	0.57	0.58
6	0.31	0.47	0.25	0.57	0.55
7	0.37	0.52	0.30	0.61	0.61
8	0.34	0.45	0.17	0.56	0.56
9	0.35	0.48	0.25	0.57	0.59
10	0.35	0.47	0.22	0.56	0.57
mean	0.35	0.46	0.23	0.55	0.56
s.e.	0.01	0.01	0.01	0.01	0.01

Both SI_gen_ and SI_epigen_ did not differ significantly between CG and OM populations (*p* = 0.245 for SI_gen_; *p* = 0.115 for SI_epigen_; table 5). Nevertheless, SI_gen_ was generally higher in CG populations, while SI_epigen_ revealed higher values in OM populations (table 5). Moreover, OM populations showed significantly higher m-subepiloci diversity (*p* = 0.035; table 5). Additionally, environmental conditions concerning light, soil moisture, soil pH, and soil nitrogen differed significantly between CG and OM populations (*p* < 0.05; table 6).

**Table 5.** Differences of genetic and epigenetic diversity (SI) between calcareous grassland and oat-grass meadow populations. Two-sided T-tests; *0.01 < *p* ≤ 0.05; n.s., *p* < 0.05. CG, calcareous grassland; OM, oat-grass meadow. h-subepiloci, CHG-hemimethylated; m-subepiloci, CG-methylated; u-subepiloci, non-methylated.

	subpopulation		*p*-value	
	CG	OM		
AFLP	0.36	0.34	0.245	n.s.
MSAP				
all subepiloci	0.45	0.48	0.115	n.s.
h-subepiloci	0.23	0.24	0.685	n.s.
m-subepiloci	0.54	0.57	0.035	*
u-subepiloci	0.54	0.58	0.067	n.s.

**Table 6.** Comparison of mean weighted EIVs between calcareous grassland and oat-grass meadow populations. Two-sided T-tests and Wilcoxon–Mann–Whitney tests; *0.01 < *p* ≤ 0.05. CG, calcareous grassland; OM, oat-grass meadow. EIV, Ellenberg indicator values of: L, light; M, soil moisture; R, soil reaction/pH; N, soil nitrogen.

	subpopulation		*p*-value
	CG	OM	
L_EIV	7.45	7.04	0.032*
M_EIV	3.43	4.86	0.012*
R_EIV	7.63	6.94	0.022*
N_EIV	2.69	5.29	0.008*

**Figure 3.**
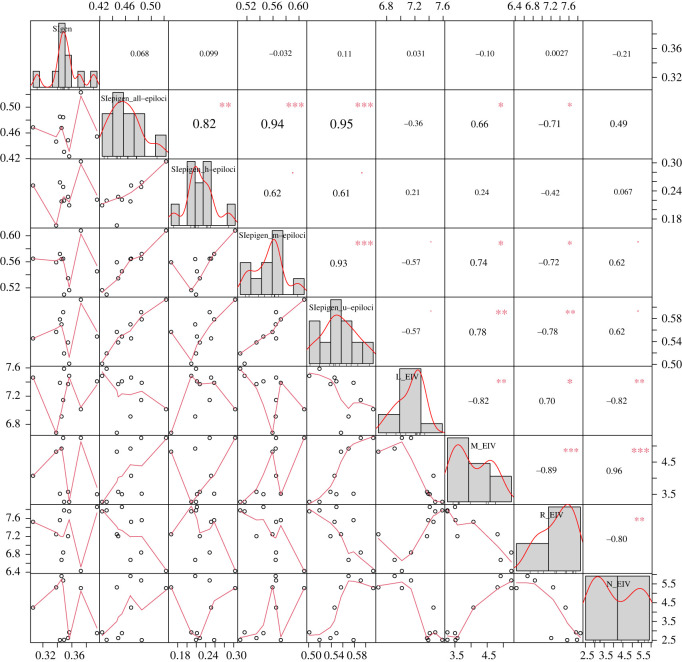
Correlation coefficients (Pearson correlation analyses) of the SI and environmental parameters represented by EIV for light (L), soil moisture (M), soil reaction/pH (R) and soil nitrogen (N).

SI_gen_ showed no correlation with environmental variables (light, soil moisture, soil pH, or soil nitrogen) (figure 3). However, SI_epigen_ (all subepiloci, m-, and u-subepiloci) was significantly associated with soil moisture and soil pH (figure 3). Thus, SI_epigen_ decreased with increasing drought and soil pH.

SI_gen_ revealed significantly lower values than SI_epigen_ (*p* < 0.001). Moreover, SI_gen_ and SI_epigen_ were not significantly correlated across populations (*ρ* = −0.13; *p* = 0.733).

## 4. Discussion

### 4.1. Genetic and epigenetic differentiation

Genetic differentiation levels were higher than epigenetic ones indicating that genetic variation may be more strongly structured than epigenetic variation [17]. Some previous studies revealed the same results [12,13], while other studies observed higher epigenetic than genetic differentiation levels [9,55,60].

Neither epigenetic nor genetic differentiation correlated with geographic distance among populations (IBD). Kloss *et al*. [61] showed that an outcrossing breeding system as well as efficient dispersal of pollen and seeds may result in similar levels of genetic diversity over large spatial scales in common grassland species. Thus, spatial isolation did not play a major role for population differentiation in *T. pratense*.

However, even common and outbreeding species may reveal increased differentiation among populations through reduced abundance, spatial isolation, different land use regimes, and thus lowered gene flow [61]. *T. pratense* populations showed higher genetic than epigenetic differentiation among habitat types. This result complies with the findings of Lele *et al*. [17], who observed that genetic variation may play a more important role in habitat differentiation than epigenetic variation. Furthermore, genetic differentiation significantly correlated with habitat dissimilarity (IBH). Reisch & Poschlod [3] observed that populations from mown and grazed habitats revealed higher genetic differentiation levels within the same geographic region than similarly managed populations among different regions in *Scabiosa columbaria*. Management practices like mowing and grazing differ strongly in intensity and time of application [3,61]. Early mowing inhibits fruiting and seed production [61], and thus mown populations may flower earlier than grazed ones [3]. These asynchronous flowering times inhibit gene flow, support genetic drift and increase, therefore, genetic differentiation levels among contrasting habitats [3]. Flowering time of *T. pratense* was shown to depend on plant weight, stem length, leaf size and further traits [62] shaped by different land use practices. Thus, land use and related gene flow patterns rather than habitat type *per se* seem to shape genetic differentiation.

### 4.2. Genetic and epigenetic diversity

Mean genetic diversity of *T. pratense* complied with genetic diversity levels previously reported for common grassland species [63]. The comparison of genetic and epigenetic diversity among contrasting habitats revealed higher genetic diversity levels in CG populations and higher epigenetic diversity levels in OM populations. These results comply with several studies, which surveyed different genetic and epigenetic diversity levels due to habitat type [2,3,9,19].

Previous studies about genetic diversity patterns in common CG [64] and OM plant species [65] observed a trend to higher genetic diversity levels in CG populations. Within the study region, CGs are still managed by migratory sheep herding and are, thus, exposed to elevated levels of disturbance by grazing and trampling. On the one hand, management-induced disturbance may create suitable niches for seeds to germinate and seedlings to establish [31]. On the other hand, grazing by sheep is an important vector for seed dispersal and enhances gene flow [66–68]. Therefore, management-related disturbance and gene flow patterns seem to increase genetic diversity levels in CG populations.

However, OM populations showed higher epigenetic diversity levels than CG populations. The difference of epigenetic diversity between CG and OM populations was significant only for m-subepiloci. Therefore, changes of methylation in the CG context (m-subepiloci) may play a more important role for habitat adjustment than regulation of gene function in the CHG context (h-subepiloci). As mentioned above, the pattern and amount of DNA methylation in plants is sensitive to biotic and abiotic stressors [23,24,69]. On the one hand, OMs represent a comparatively homogeneous habitat type with narrow ecological niches, since all species are simultaneously disturbed by mowing. Previous studies showed that an increase in epigenetic diversity may broaden ecological niches by expanding the species' potential to resist disturbance events [60,70]. On the other hand, Pearson correlation analyses indicated that epigenetic diversity of *T. pratense* populations significantly decreased with increasing drought and soil pH. Therefore, challenging environmental conditions may affect epigenetic diversity in different ways.

Pearson correlation revealed no significant association between genetic diversity and environment. Pagel *et al*. [65] postulated landscape structure as a key variable for genetic diversity of *T. pratense* populations in OMs, while they could not observe any impact of local habitat quality. Therefore, genetic diversity of *T. pratense* may be affected more by landscape structure, related management, and/or gene flow patterns than by local environmental conditions.

However, several studies reported correlations between environmental factors and epigenetic characteristics of plant populations [9,12,13]. In this study, epigenetic diversity correlated significantly with soil moisture and soil pH. Thus, the epigenetic diversity of *T. pratense* populations seemed to be associated with environment, while genetic diversity was not. These results accompany the assumption that DNA methylation and demethylation at a genome-wide scale are induced by environmental changes [9] and constitute an essential tool for plant species to react on biotic and abiotic environmental pressures [24,69]. Moreover, epigenetic variation is supposed to increase under challenging environmental conditions [20–22]. Labra *et al*. [24] emphasized that different plant species may show varying DNA methylation patterns depending on the kind of challenging environmental conditions. Thus, the assumption that epigenetic diversity grows under challenging environmental conditions should not be generalized across all species. In this study, epigenetic diversity decreased under drought. This result was in line with the study of Davis [71], who observed that *T. pratense* produced less yield under drought stress. Furthermore, Labra *et al*. [24] postulated that active methylation or demethylation of cytosine could occur dynamically in response to water stress [20]. Thus, epigenetic diversity of *T. pratense* populations may decrease with increasing drought. Additionally, epigenetic diversity decreased with increasing soil pH. Soil pH influences the amount of plant available nutrients. Since *T. pratense* is a nitrogen-fixing legume [38], its performance is sometimes limited by plant accessible phosphorus [71]. In calcareous soils, phosphorus is bound to calcium phosphate [72] and thus not plant available. The CGs in our study revealed the highest soil pH. Therefore, *T. pratense* populations may show limited productivity and decreased epigenetic diversity as reaction to phosphorus limitation. However, the correlation with soil moisture and soil pH was not significant for h-epiloci indicating that the regulation of gene function by (de-)methylation in the CHG context may not be an issue for adaptation to different environmental conditions.

Previous studies observed higher levels of epigenetic than genetic diversity especially in natural plant populations [9,13,17,18]. In *T. pratense*, epigenetic diversity was even significantly higher than genetic diversity indicating that these natural populations seem to vary more in DNA methylation than in DNA sequence [73].

Furthermore, neither correlation nor simple Mantel tests revealed a significant association of epigenetic with genetic diversity or distance. In this context, Richards [28] defined three classes of epigenetic variation at a given locus: (i) obligatory: epigenotype is strictly determined by genotype, (ii) facilitated: epigenotype depends on both genotype and environmental context, or (iii) pure: epigenotype is created by environmental context. On the one hand, Foust *et al*. [13] stated that studies which cannot sample the entire genome may miss genomic elements or genes that are involved in or affected by DNA methylation. On the other hand, they considered the application of molecular markers in natural populations as a useful tool to identify epigenetic structures, which are not explained by DNA sequence. Thus, we assume that epigenetic and genetic diversity may differ in their ecological and evolutionary implications [18,74] and classify the epigenetic variation of *T. pratense* populations as facilitated or pure rather than obligatory. This finding is in accordance with the results of previous studies on wild plants, which also observed epigenetic variation to be largely autonomous from genetic variation [10,75].

## 5. Conclusion

Our results revealed an impact of different environmental conditions on genetic and epigenetic variation. Genetic variation was affected by habitat-specific environmental conditions induced by management-related disturbance as well as gene flow patterns. Epigenetic variation was driven by challenging environmental conditions in two ways. It increased with rising necessity for niche establishment, but decreased under drought and high pH, the latter potentially resulting in phosphorus limitation.

Nevertheless, MSAP markers reveal only a limited number of anonymous loci, which are difficult to link to functional genomic elements. Therefore, future studies should apply next-generation-based bisulfite sequencing approaches to evaluate the effects of challenging environmental conditions on methylation patterns more precisely [17].

## Data Availability

All data generated or analysed during this study are included in this published article and its electronic supplementary information file [76].
